# A Case Report of Angiotensin II Use in the Treatment of Refractory Shock due to Amlodipine and Lisinopril Toxicity

**DOI:** 10.1155/crcc/7543758

**Published:** 2024-12-11

**Authors:** John H. Keller, Kayla J. Kendric, Kathy T. LeSaint

**Affiliations:** ^1^Department of Emergency Medicine, University of California San Francisco, San Francisco, California, USA; ^2^California Poison Control System, San Francisco Division, San Francisco, California, USA

**Keywords:** ACE inhibitor, angiotensin II, angiotensin-converting enzyme inhibitor, case report

## Abstract

**Introduction:** Coingestion of cardiovascular drugs with angiotensin-converting enzyme inhibitors (ACEIs) can be associated with refractory shock derangements complicated by vasopressor resistance, prompting the use of novel, unconventional, or uncommonly used agents.

**Case Report:** A young adult male presented to the emergency department (ED) 10 h after ingesting lisinopril and amlodipine. On arrival, he was hypotensive with a blood pressure of 72/39 mmHg. In addition to crystalloid fluids, he was incrementally started on four vasopressors including norepinephrine, phenylephrine, epinephrine, and vasopressin without improvement in mean arterial pressure (MAP). He was then administered methylene blue, calcium gluconate, and hyperinsulinemic euglycemia therapy after discussion with medical toxicology. Shortly afterwards, he was started on an angiotensin II infusion with an improvement in MAP to a goal of > 65 mmHg.

**Conclusion:** Despite evidence of efficacy in refractory vasodilatory shock secondary to sepsis, there is a paucity of data on the use of angiotensin II as an adjunctive vasopressor in drug-induced shock. We report a case of successful use of angiotensin II in combined lisinopril and amlodipine overdose refractory to conventional vasopressor support. Combined overdose of ACEIs with calcium channel blockers (CCBs) has been shown to cause more significant hypotension and higher vasopressor requirements than overdose of CCBs alone. This may be due to the synergism between CCBs and ACEIs, where the normal homeostatic mechanism of the renin–angiotensin–aldosterone system (RAAS) activation in response to shock is now inhibited, leading to decreased compensatory vasoconstriction via angiotensin II and decreased endogenous catecholamine release. We hypothesize that angiotensin II may have been of particular benefit in this patient given the likelihood that reduced angiotensin II levels were contributing to his refractory shock.

## 1. Introduction

Cardiovascular drugs are among the substances most frequently involved in human poisoning exposures. According to the America's Poison Centers' National Poison Data System, cases with serious outcomes increased significantly in the last decade [1]. Of the substance categories associated with the largest number of fatalities, angiotensin-converting enzyme inhibitors (ACEIs) are among the top 25 [1].

ACEIs inhibit angiotensin–converting enzymes which prevent the conversion of angiotensin I to angiotensin II [2]. Angiotensin II is a peptide hormone in the renin–angiotensin–aldosterone system (RAAS) with several clinical effects, including arteriolar vasoconstriction and increased sympathetic outflow from the CNS [3]. Overdoses of ACEIs, however, typically do not cause hemodynamic instability though coingestion with other cardiovascular drugs can be associated with refractory shock and significant metabolic derangements [4]. In the case of coingestion, treatment of vasoplegic shock may be challenging and complicated by vasopressor resistance, prompting the use of novel, unconventional, or uncommonly used agents. In this report, we describe a case of angiotensin II (Giapreza, La Jolla Pharmaceuticals) used in the treatment of concomitant ACEI and calcium channel blocker (CCB) poisoning with subsequent improvement in hemodynamic status.

## 2. Case Report

A young adult male with a past medical history significant for untreated depression, attention-deficit/hyperactivity disorder previously on amphetamine and dextroamphetamine (Adderall), alcohol use, and marijuana use presented to the emergency department (ED) with the chief complaint of drug overdose. Per first responders, the patient had ingested his father's medications approximately 10 h prior to arrival: 20 tablets of lisinopril (20 mg) and 20 tablets of amlodipine (10 mg). En route to the ED, he was reportedly hypotensive, though no blood pressure was documented, and given 1200 mL of normal saline before arrival without significant improvement.

On arrival to the ED, his vital signs were as follows: temperature of 37.1°C, blood pressure of 72/39 mmHg, heart rate of 108 beats/minute, respiratory rate of 18 breaths/minute, and oxygen saturation of 99% on room air. His physical exam was notable only for tachycardia. He was alert and conversant. A complete blood count and comprehensive metabolic panel were remarkable for leukocytosis (white blood cell: 14.6 K/uL; normal: 4.0–11.0 K/uL) and kidney injury (creatinine: 2.94 mg/dL; normal: 0.67–1.17 mg/dL). A urine drug screen was positive only for tetrahydrocannabinol. Acetaminophen level, acetylsalicylic acid level, brain natriuretic peptide, coagulation panel, ethanol level, lactate, liver function tests, thyroid stimulating hormone, and urinalysis were unremarkable. An electrocardiogram showed normal sinus tachycardia with normal axis, normal intervals, and no evidence of cardiac ischemia. The patient was started on a 30 cc/kg infusion of crystalloid fluid, a norepinephrine infusion, and subsequent concomitant phenylephrine, epinephrine, and vasopressin infusions after his blood pressure did not improve (mean arterial pressure (MAP) remained at 50–60 mmHg). During his ED course, he also became intermittently agitated, requiring a single dose of midazolam (2 mg) and two doses of haloperidol (5 mg). He was admitted to the medical intensive care unit (MICU) for refractory distributive shock.

In the MICU, a bedside echocardiogram revealed a “grossly normal ejection fraction.” The patient's four vasopressors were uptitrated to “maximum” doses: norepinephrine at 0.016 mg/mL, phenylephrine at 0.16 mg/mL, epinephrine at 0.016 mg/mL, and vasopressin at 0.4 units/mL. Additionally, he was given methylene blue (70 mg) and 3 doses of calcium gluconate (1 g), and hyperinsulinemic euglycemia therapy (HIET) was initiated at 2 units/kg/hour. Extracorporeal treatment was considered but was not instituted due to hemodynamic instability. For possible sepsis, he was started on vancomycin (1 g) and cefepime (2 g) as broad-spectrum antibiotic coverage.

Approximately 8 h after arrival to the ED, the patient was initiated on an angiotensin II infusion at 4 ng/kg/min, and the patient's blood pressure subsequently improved to a maximum blood pressure of 106/62 mmHg, meeting a MAP goal of > 65 mmHg. Over 2 days, epinephrine and vasopressin were titrated off, followed by norepinephrine and phenylephrine on hospital day 3. On hospital day 4, both HIET and angiotensin II were titrated off with subsequent measured MAPs in the 70s mmHg.

During the patient's hospital course, he had persistent agitation, delusions, and paranoia requiring a dexmedetomidine infusion. A psychiatry consultation team diagnosed the patient with new-onset schizophrenia. He was placed on a psychiatric hold and initiated on oral valproic acid and haloperidol for agitation/anxiety. Dexmedetomidine was weaned off on hospital day 6. He was deemed medically stable and transferred to the inpatient psychiatric unit on hospital day 7. The patient has been deidentified for this publication.

## 3. Discussion

Despite evidence of efficacy in refractory vasodilatory shock secondary to sepsis, there is a paucity of data on the use of angiotensin II as an adjunctive vasopressor in drug-induced shock [5]. We report a successful use of angiotensin II in combined lisinopril and amlodipine overdose refractory to conventional vasopressor support. Despite ACEIs being well tolerated in isolated ingestions, combined overdose of ACEIs with CCBs has been shown to cause more significant hypotension and higher vasopressor requirements than overdose of CCBs alone [6]. This may be due to the synergism between CCBs and ACEIs, where the normal homeostatic mechanism of RAAS activation in response to shock is now inhibited, leading to decreased compensatory vasoconstriction via angiotensin II and decreased endogenous catecholamine release [7]. Beyond inhibiting the synthesis of angiotensin II, ACEIs also block the breakdown of bradykinin into inactive peptides. This results in bradykinin accumulation, which promotes vasodilation and likely contributes to the hypotension observed in overdose [8].

Angiotensin II (Giapreza, La Jolla Pharmaceuticals) was approved by the Federal Drug and Administration (FDA) in 2017 for intravenous use for the indication of vasoconstriction in adults with distributive shock. Angiotensin II is a naturally occurring peptide hormone of the RAAS. In the RAAS, renal afferent arteriole cells synthesize the proteolytic enzyme renin. Decreased arterial blood pressure leads to the release of active renin into the systemic circulation and surrounding tissues. Renin release allows for angiotensinogen production, which later converts angiotensin I, a decapeptide with weak biological activity, to angiotensin II. Angiotensin II enacts its strong vasopressor activity through rapid degradation to angiotensin III and IV, which subsequently bind and interact with specific G protein-coupled receptors including angiotensin receptor I. This stimulates Ca2+/calmodulin-dependent phosphorylation of myosin and causes smooth muscle contraction which results in vasoconstriction. Additionally, angiotensin II increases blood pressure by stimulating aldosterone release by the adrenal zona glomerulosa, increasing sodium and water reabsorption in the proximal tubular cells, and increasing vasopressin release.

In the ATHOS-3 clinical trial, 70% of patients treated with angiotensin II reached the target MAP at hour three after administration compared to 23% of placebo subjects, a statistically significant treatment effect of 47% [9]. In the angiotensin II group, the MAP increased rapidly, allowing the angiotensin II dose to be decreased from the original 20 ng/kg/min in 67% of the patients within 30 min and permitting decreases in doses of concomitant vasopressors [9]. The median time to reach the target MAP endpoint was 5 min in the angiotensin II treatment group [9]. In a systematic review of 14 reported cases where angiotensin II was used in poisoning-induced shock, three patients (30%) had an immediate improvement in MAP or SBP after initiation of angiotensin II, four patients (40%) had responses within 30 min, and one patient (10%) had a response within 2 h [10]. While the authors did not conduct a subset analysis of its effectiveness for specific drug classes, it is noteworthy that all 7 of the patients who overdosed on an ACEI (with or without coingestants) experienced a prompt improvement in blood pressure following angiotensin II administration.

We hypothesize that angiotensin II may have been particularly beneficial in this patient due to the likely contribution of reduced angiotensin II levels to his refractory shock. This case has a few limitations. Notably, the exact time of angiotensin II initiation and the MAP at that time are unknown, though it was recorded that the patient had not yet reached the goal MAP of > 65 mmHg when angiotensin II was started. Additionally, the precise time to MAP improvement following angiotensin II administration is unclear, although the documentation indicates that MAP did improve afterward. The patient received multiple medications that could have influenced MAP, including catecholamines, vasopressin, methylene blue, and high-dose insulin, which may have contributed to his clinical improvement. However, the chart notes that the patient remained in refractory shock despite the administration of catecholamines and vasopressin. Despite these limitations, our case report supports this growing body of evidence for the use of angiotensin II in drug-induced shock, particularly when an ACEI is implicated.

## 4. Conclusion

Isolated ACE inhibitor overdoses are typically well tolerated, but coingestion with other cardioactive substances can lead to severe, refractory shock. Angiotensin II may benefit these patients by bypassing the inhibition of the RAAS caused by ACE inhibitors.

## Data Availability

The data that support the findings of this study are available on request from the corresponding author. The data are not publicly available due to privacy or ethical restrictions.
